# Rapid Detection of Rongalite via a Sandwich Lateral Flow Strip Assay Using a Pair of Aptamers

**DOI:** 10.1186/s11671-018-2709-9

**Published:** 2018-09-24

**Authors:** Jie Li, Le Jing, Yuzhu Song, Jinyang Zhang, Qiang Chen, Binghui Wang, Xueshan Xia, Qinqin Han

**Affiliations:** Engineering Research Center for Molecular Diagnosis, Faculty of Life Science and Technology, Kunming University of Science and Technology, Kunming, 650500 Yunnan People’s Republic of China

**Keywords:** Aptamer, AuNPs, Lateral flow strip, Detection, Rongalite

## Abstract

A sandwich lateral flow strip assay (LFSA) using a couple of aptamers functionalized with gold nanoparticles (AuNPs) was designed to assess the presence of rongalite in agrifood products. More specifically, a biotin-labeled primary A09 aptamer immobilized on a streptavidin-coated membrane and a secondary B09 aptamer conjugated with AuNPs were developed as capturing and signaling probes, respectively. This system allows the successful and direct detection of rongalite in food samples with concentrations as low as 1 μg/mL, simply by observing the color change of LFSA control and test line.

## Background

Rongalite (sodium hydroxymethylsulfinate) is an industrial reagent typically used for vat dyeing [1] or for emulsion polymerization [2] as a reducing agent. Rongalite may also be found in water conditioner (e.g., reduction of chlorine and chloramine) [3], in commercial cosmetic hair color removers despite the generation of formaldehyde (a known human carcinogen), or even in pharmaceutical formulations as an antioxidant [4]. This compound also caused adverse effects in China after its incorporation in several agrifood products [5]. This developed assay provides a reliable on-site rongalite detection platform and can contribute to solve food security issues.

Aptamers, single-stranded oligonucleotides, and oligopeptides, have been considered as perfect alternatives to antibodies owing to their high specificity, easy and reproducible production, easy modification, and less immunogenic response [6]. Recent studies have revealed the strong potential of aptamers as bioprobes for drug targeting, biosensing, and the development of new drugs [7]. Electrochemical [8] and enzyme-linked aptamer [9] assays involving a couple of aptamers have been developed as a promising tool for rongalite detection. However, these methods typically suffer from long analysis times and complex procedures, which hinder their applications [10].

Lateral flow strip assay (LFSA, also called strip test) was first developed in 1956 as a logical extension of the latex agglutination test technology [11]. As a single-step approach, LFSA has attracted significant attention for the on-site detection of multiple analytes due to its user-friendly format, low production cost, time-saving use, and long-term stability over a broad range of conditions [12].Despite these positive characteristics, the practical applications of the aptamer-based lateral flow strip platform have not been commercialized yet, and only a few aptamer-based chromatographic strip assays have been reported for the detection of rongalite in food samples [13]. In view of the high occurrence of food security affairs and the common use of rongalite as an illegal food additive, it is necessary to develop an aptamer-based LFSA for the on-site and rapid detection of this compound in food samples.

Two types of lateral flow strip aptamer sensors can be developed, namely, competitive and sandwich-type [14]. The sandwich-type platform is highly suitable when a couple of aptamers are available for a specific target molecule. In the present work, specific gold nanoparticles (AuNPs), which are known to be the most promising nanomaterials for aptamer sensor development (e.g., physico-chemical properties), were employed for the development of a lateral flow sandwich strip aptamer-detecting probe. Meanwhile, aptamer conjugation processes have been previously demonstrated on AuNPs via chemisorption or physical adsorption which provides a simple yet sensitive platform for the aptamer sensor which was later used as a signaling probe in this study [15]. Owing to the advantages derived from the use of AuNPs and aptamers, a visible, rapid, one-step, and on-site lateral flow assay was developed for the analysis of rongalite in food samples. In order to achieve this sandwich-type aptamer sensor, two aptamer probes (A09/B09) were used serving as capturing and signaling probes. The positive results of this biosensor were further confirmed by high performance liquid chromatography (HPLC).

## Methods

### Materials and Reagents

Rongalite was provided by Tokyo Chemical Industry Co., Ltd. HAuCl_4_ (trisodium citrate dehydrate) was purchased from Sigma Aldrich (USA). NaCl, BSA, sucrose, formalin, PEG20000, and Tween20 were from Beijing Biotopped Science and Technology Co., Ltd.

NC membranes (i.e., pall 90, pall 170, and Millipore 135) are from Pall Corporation and Millipore Corporation, separately, and purchased from Jiening Biotech Company.

Food samples, ersi (thin-cut square strands of rice cake in China), noodles, tofu, and glucono-δ-lactone-tofu, were purchased from the nearby markets.

### Aptamer Preparation via Systematic Evolution of Ligands by Exponential Enrichment (SELEX)

**Fig. 1:**
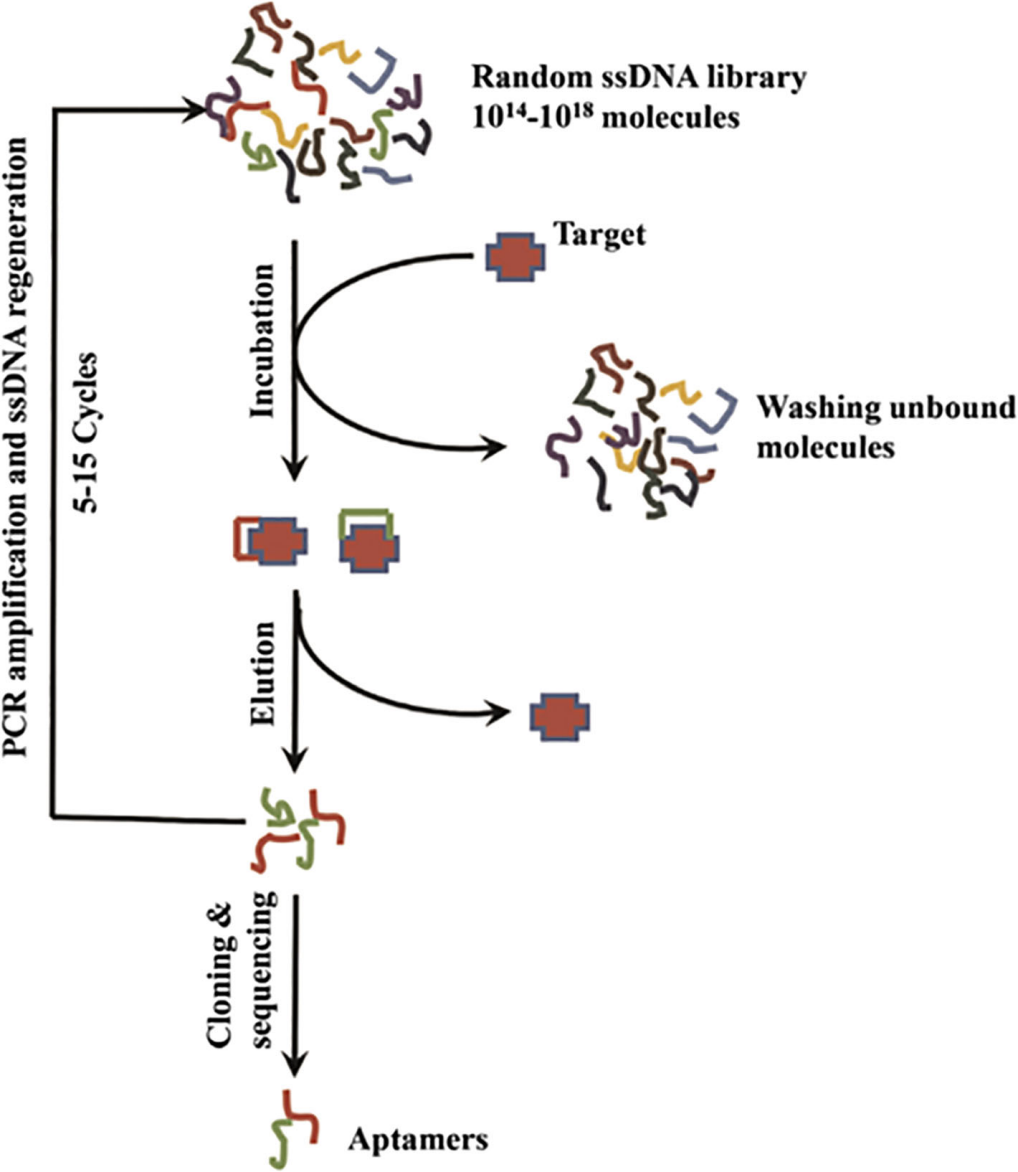
Typical aptamer selection (SELEX) process

The SELEX process comprises the following steps (Fig. 1): (i) incubation of the target with a random DNA library, (ii) isolation of the DNA bound to the target, (iii) DNA amplification by polymerase chain reaction (PCR), (iv) preparation of single-stranded DNAs for the next-round library, (v) repetition of steps i–iv for 5–15 cycles involving interval counter screening with non-target substances to remove the nonspecific ssDNAs, and (vi) final cloning and sequencing of the enriched library [16].

The aptamers were screened following the steps described previously. Briefly, an initial ssDNA aptamer library consisting of 82-mer nucleotides with central 40-based long randomized sequence of each aptamer was synthesized (Tsingke Biological Technology). The randomized sequence of the ssDNA aptamer pool is 5′-GACATATTCAGTCTGACAGCG-N40-GATGGACGAATATCGTCTAGC-3′, where N means a randomized nucleotide of either A, G, C, or T.

In this research, primer 1 (5′-GACATATTCAGTCTGACAGC-3′) and primer 2 (5′-GCTAGACGATATTCGTCCATC-3′) were used for amplification of the library.

To screen aptamers specifically binding to rongalite, the synthesized random ssDNA library was added to the plate with rongalite. Next, the unbound ssDNA was removed by washing; the bound ssDNA was recovered and amplified by PCR. The binding affinity of aptamers gradually increased as the selection round increased.

The specificity and *K*_d_ value of individual aptamers was determined similarly to Tang’s methods [17].

The aptamer sequences used in this work were as follows:

- Primary aptamer A09,
  5′-Biotin-GACATATTCAGTCTGACAGCGGAAGCGGGTCAGTCCAACTCACGGTCTCGCATGCACGGGAGATGGACGAATATCGTCTAGC
- Secondary aptamer B09,
  5′-Biotin-GCTAGACGATATTCGTCCATCTCCCGTGCATGCGAGACCGTGAGTTGGACTGACCCGCTTCCGCTGTCAGACTGAATATGTC-HS-3′

These aptamer sequences (A09 and B09) were synthesized and purchased from Tsingke Biological Technology.

### Production of AuNPs

**Fig. 2:**
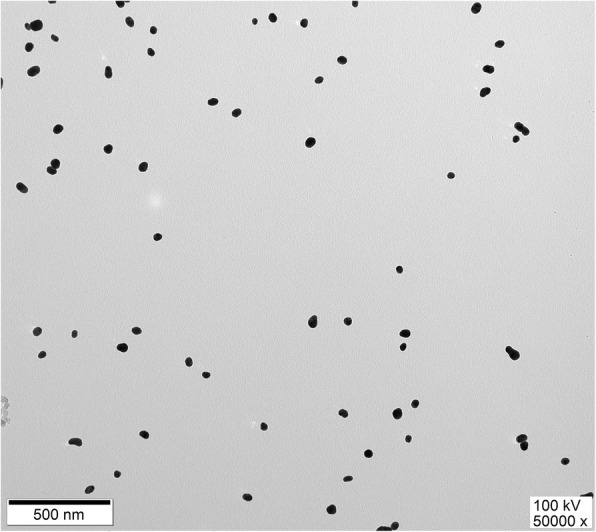
TEM images of AuNPs (40 nm)

AuNPs were synthesized via the citrate reduction of HAuCl_4_ protocol [18]. Monodispersed production of AuNPs with adequate size was confirmed by UV/Vis spectrophotometry (Thermo Scientific™ Evolution 60S). Spherically shaped (ca. 40 nm in diameter) and deep red colored AuNPs were synthesized. The size of these unmodified AuNPs was estimated by using the Beer–Lambert law at 530 ± 2 nm and transmission electron microscopy (Fig. 2) (JEOL Ltd. JEM-1011).

The secondary aptamer B09 conjugated with AuNPs was prepared by following reported protocols [18]. Briefly, 1 mL of the as-prepared AuNPs was incubated with 4 μL of the secondary aptamer B09 solution (100 μM), and the mixture was stored in a drawer at room temperature for at least 16 h. One molar of NaCl was subsequently added dropwise to the vial upon gentle hand shaking until reaching a final concentration of 0.1 M in the mixture. The vials were stored in a drawer for at least 1 day before use. The unconjugated thiolated aptamers were removed by centrifugation at 12,000*g* for 20 min at 4 °C. The tubes were removed from the centrifuge and a clear supernatant liquid was obtained, with the nanoparticles being at the bottom of the tubes. The mixture was centrifuged for additional 5 min in case of red color supernatant. The supernatant was gently pipetted off and the nanoparticles were finally dispersed in 1 mL of 0.01 M PBS (phosphate-buffered saline) buffer (pH = 7.4) containing 5% BSA, 5% sucrose, 1% PEG20000, and 0.05% Tween20.

### Lateral Flow Strip Design

**Fig. 3:**
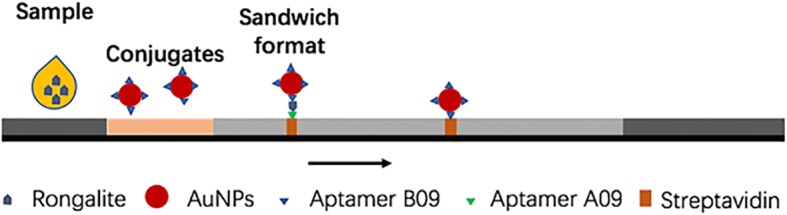
Typical lateral flow test strip configuration (sandwich format)

The LFSA was designed as shown in Fig. 3. Briefly, the strip contained several overlapping pads on a backing card sample (GF-08, 20 × 300 mm), gold conjugate, nitrocellulose (NC) membrane (Pall 90, 60 × 300 mm), and absorption (H-5076, 20 × 300 mm) pads. The length of the overlapping was 2 mm. The sample pad was immersed in a 0.01 M PBS buffer (pH = 7.4) containing 3% BSA and 0.05% Tween20 and subsequently dried at 37 °C for 2 h. The gold conjugate pad was treated with a 0.01 M PBS buffer (pH = 7.4) containing 5% BSA, 5% sucrose, 1% PEG20000, and 0.05% Tween20. The functional AuNPs were subsequently sprayed with a sprayer (XYZ3010-1429) on the treated gold conjugate pad. One microliter of biotin-A09 aptamer (10 μM) was incubated with 10 μL of streptavidin (1 mg/mL) for 1 h at 4 °C. The aptamer-conjugated streptavidin was subsequently lined with a dispenser (XYZ3010-1429) on a NC membrane to form the test line, while streptavidin (1 mg/mL) was lined with the instrument to form the control line. After that, the treated NC membrane was dried at 37 °C for 2 h for immobilization. Finally, the strips were assembled and the LFSA was cut into 40-mm wide strips and stored at 37 °C overnight until use.

### Specificity Test

Eighty microliters of a rongalite solution (10 μg/mL) was added to the sample pad of the assembled strips. This step was repeated for the other counter targets including formalin and deionized water for the specificity tests. Deionized water was used as a control. A red line should appear in the expected lined area. Each control was repeated twice.

### Dose-Dependent Test

Similar to the specific test, rongalite solutions with varying concentrations (0.8, 1, 5, and 10 μg/mL) were prepared. Eighty microliters of the rongalite solution was added to the sample pad of the assembled strips. The observation of red color within 15 min on the test line was regarded as the criteria for determining the detection limit.

### Food Sample Test

To evaluate the practicability and accuracy of this novel LFSA, five food samples possibly containing added rongalite were collected form a market around the institute. One gram of sample was extracted with 10 mL of water. Then, 80 μL of each sample extract solution was applied to the aptamer-based lateral flow strip for the detection of rongalite. These results were confirmed by high-performance liquid chromatography (HPLC).

## Results and Discussion

### The Specificity and Dissociation Constants (*K*_d_) of the Selected Aptamers

**Fig. 4:**
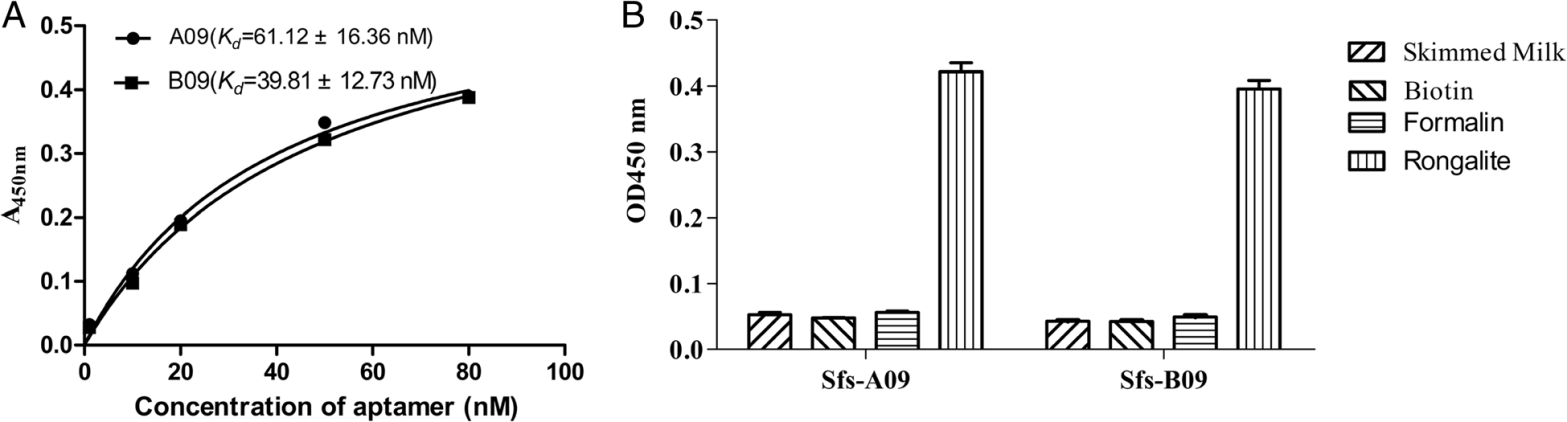
**a** Measurement of the *K*_d_ value of A09 and B09. GraphPad Prism was used to perform nonlinear cure fitting analysis for *K*_d_ calculation. **b** The specific binding affinity between A09/B09 and rongalite

Different concentrations of A09 and B09 aptamers were incubated with a fixed amount of rongalite. Saturation curves plotting the measured absorbance at 450 nm against the corresponding input aptamer concentration are shown in Fig. 4a. Non-linear regression analysis was used for *K*_d_ value calculation. The *K*_d_ value of A09 is 61.12 ± 16.36 nM and B09 is 39.81 ± 12.73 nM. As shown in Fig. 4b, the binding affinity between A09/B09 and rongalite is high.

### Specificity and Sensitivity

The aptamer A09 labeled with biotin (capturing aptamer) was bound to streptavidin initially lined on the membrane. This line was selected as the test line. Meanwhile, streptavidin was aligned on the control zone.

**Fig. 5:**
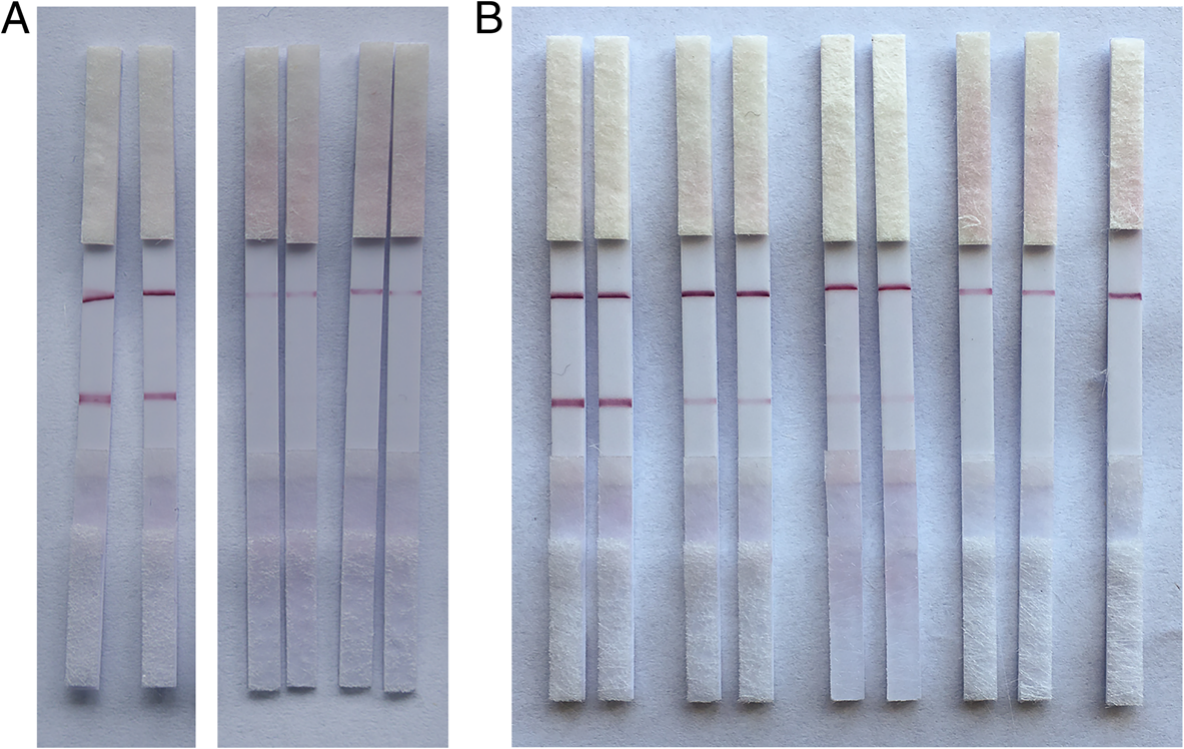
Specificity (**a**) and sensitivity (**b**) of LFSA for rongalite. **a** 80 μL of a rongalite solution (10 μg/mL) was added to the sample pad of the assembled strips. This step was repeated for the other counter targets including formalin for the specificity tests. Deionized water was used as a control. **b** Rongalite standard solutions with varying concentrations (0, 0.8, 1, 5, and 10 μg/mL) were prepared. 80 μL of the standard solutions was pipetted to the sample pad, and the observation of red color within 15 min on the test line was regarded as the criteria for determining the detection limit

As shown in Fig. 5a, once the AuNP secondary aptamer (as a signaling probe) is bound to rongalite, the primary aptamer lined on the test zone is bounded to another site of this compound. A red line generated by AuNPs should appear on the test zone in case of positive analysis. With regard to the control experiment, the streptavidin on the control zone captures the remaining AuNP-labeled B09 aptamer modified with biotin, thereby providing a control signal at all times.

As shown in Fig. 5b, the result shows rongalite was easily detected with the naked eye at concentrations as low as 1 μg/mL.

These food samples were analyzed via the herein developed LFSA, and the results are shown in Table 1.

**Table 1:** Rongalite detected in real samples by aptamer-based lateral flow strip and HPLC

Number of sample	LFSA (*n* = 3)	HPLC
	Visual results	
S1	− − −	−
S2	− − −	−
S3	− − −	−
S4	u u u	+
S5	+ + +	+

In sample 4, glucono-δ-lactone-tofu solution, the control line is absent, but in sample 5, glucono-δ-lactone-tofu, a positive result appears. u means the results are useless while in the control zone a red line does not appear

Interestingly, the control line was not maintained after each LFSA. High concentrations of rongalite or salt ions can result in a faint signal on the control zone. Therefore, the composition of the re-suspending buffer affects critically the performance of the strip test. According to the obtained results, a 0.01 M PBS buffer (pH = 7.4) containing 5% BSA, 5% sucrose, 1% PEG20000, and 0.05% Tween20 was chosen as the re-suspending buffer.

The composition of the various pads has a dramatic effect on the performance of the strip assay. Among the various alternatives, NC membrane was found to be the most suitable solid support for the adsorption and hybridization of nucleic acids. NC has been widely used as a signal pad in lateral flow strip since it provides sufficient flow rates [19]. Different size types of NC membranes with respective flow rates can be suitable for these assays. In this work, three commonly used NC membranes (i.e., pall 90, pall 170, and Millipore 135) purchased from Jiening Biotech Company were tested. Pall 90 was chosen herein as the most suitable NC membrane for LFSA.

A numerous number of technologies had been developed for rongalite detection. However, few have been widely applied in the on-site detection, primarily because of the associated high costs and complex protocols, such as GC and HPLC, which are cumbersome for the daily operator. LFSA, a single-step approach, has become a perfect platform owing to its user-friendly format, low production cost, and convenience. Despite the worse sensitivity than chromatograph strips, LFSA would be a promising method in point-of-care testing field.

LFSA has still a lower sensitivity than chromatograph strips. In addition, LFSA technologies using aptamers show some inherent advantages over lateral flow immunoassay (LFIA, antibody-based method) and this regardless of the recent advances in this field. Although similar assays can be also designed using antibodies, aptamer sensors offer stability and low-cost advantages. Besides, aptamers are more flexible for developing different formats since they are composed of nucleic acids having intra- and inter-molecular hybridization, enzymatic replication, and easy sequence determination characteristics. In virtue of these positive properties, numerous aptamer sensors have been developed for multiplexed assays.

Additionally, LFSA can use different labels including recently developed quantum dots [20] and upconverting phosphors [21]. However, among all reported labels, AuNPs are the most widely used for LFSA. The most remarkable property of the Au label lies in its ability to color the NC membrane allowing direct observation by the naked eye. This characteristic differentiates LFSA from current expensive laboratory methods making this technology a convenient analytic tool.

Point-of-care testing (POCT) has been proposed as an ideal tool to reduce the costs of these assays. The LFSA biosensor platform, the most widely known assay, is currently used for POCT [22]. The LFIA biosensor platform mainly includes sandwich and competitive (or inhibition) formats. In general, the sandwich format assays are designed in case of target molecules having at least two epitopes. A dual aptamer bounded to rongalite at two different binding sites was developed herein containing capturing and signaling probes assembled in the sandwich-type format.

## Conclusions

An easy and low-cost LFSA with a sandwich format was successfully developed for on-site rapid detection of rongalite. The assay involved a couple of aptamers conjugated with AuNPs. After optimizing some key parameters, the developed assay provided a high sensitivity with detection limit values as low as 1 μg/mL. This technology could be easily used for studying the contamination of food samples with rongalite. This assay provides a reliable on-site rongalite detection platform and can contribute to solve food security issues.

## Abbreviations

AuNPs: Gold nanoparticles
HPLC: High-performance liquid chromatography
LFIA: Lateral flow immunoassay
LFSA: Lateral flow strip assay
NC membrane: Nitrocellulose membrane
POCT: Point-of-care testing
SELEX: Systematic evolution of ligands by exponential enrichment
TEM: Transmission electron microscopy
